# Clinical Cases: Cannabis—In Effects of Post-partum Hemorrhage; Acid. Mur.—Diarrhœa; Natr. M., Canth., Rhus Tox. and Merc.—Gleet

**Published:** 1881-09

**Authors:** E. W. Berridge

**Affiliations:** London


### CLINICAL CASES.

*Cannabis*—In the effects of post-partum hemorrhage.

*Acid. Mur.*—Diarrhœa; *Nat. m.*, *Canth.*, *Rhus tox.* and *Merc.*—Gleet.

E. W. Berridge, M. D., London.

*Cannabis in effects of post-partum hemorrhage.*—June 28th, 1870. Mrs. —— was confined on May 15th, and had much hemorrhage; since then has had the following symptoms: very weak; giddiness when walking, with feeling of falling (once she actually fell forward), and at the same time feeling as if she would lose her senses; every day pain in right temple, and vertex as if opening and shutting, beginning on waking and lasting all day, off and on, worse from noise; head feels as if it would fall in all directions; for the last week (this is the *latest* symptom) voices, including her own, seem to come from a distance; her own voice seems strange, as if it were some one else speaking from a distance; memory bad, forgets when speaking what she is going to say; forgets what she has to do, if she does not make a note of it; appetite poor, dislikes meat, of which she is naturally fond; time seems prolonged, especially for the last week or two; every day faint feeling, sometimes faints right off; cannot follow long what people say to her, seems to be in a dream, as if things were not real (this is one of the earliest symptoms); feels at times as if she were somebody else; sometimes feels as if she did not know where she was, objects seem strange; disagreeable taste in mouth on waking, disappearing after cleansing teeth, but returning after meals; when writing, repeats or omits words; after looking long, mistiness before eyes, so that she cannot see well.

*Diagnosis of the remedy.*—The latest symptom is always (*cæteris paribus*) the most important in the choice of the remedy. In the present case, this symptom was "voices seem to come from a distance," which is found under *Cann. ind.*, *Cham.*, *Erythrox.*, *Nitrogen-oxyg.*, *Petiv.* and *Solan. nig.* (Compare also *Alum.* and *Carb. an.*) The most *peculiar* symptom was the pain in head, as if opening and shutting; this symptom is found only under *Cann. ind.* I have verified it in another case, and a colleague has informed me that *Cann. sat.* greatly relieved the same feeling in the back, thus showing that the *character* of a symptom may be the key-note of a case, and that a medicine may cure a very peculiar symptom, even though it has not as yet been produced in the same anatomical region, but only in another, as I have myself verified. (According to a late proving, *Natr. hypochloros.* has "feeling as of opening and shutting in the womb;" but this is hardly the same symptom as when occurring in a hard, non-expansive organ; and, moreover, it does not otherwise correspond. See also *Actæ racem.* in Hering's "Guiding Symptoms," p. 44.) The list is therefore at once reduced to *Cann. ind.*, which also corresponds to the large majority of the remaining symptoms, many of these having been elicited by my own published provings. The question here naturally arises—are *Cannabis Indica* and *Sativa* the same species; and if the same, botanically, do the different conditions of climate under which they grow, give them different medicinal properties? Provings *alone* will never decide this question, as the differences in the pathogeneses might depend upon idiosyncrasies of the provers; but careful clinical observations combined with provings can determine it. As a help to the solution of the problem, I gave the patient one dose of *Cann. sat.*, 1000 (Jenichen).

June 29th, 8.30 P. M. This morning in better spirits and looked better; not quite so well this evening, owing to extra fatigue; feels stronger; less giddiness in morning, but it returned in evening with feeling as if she would fall forwards; no feeling of losing senses; no pain in head on waking, it came on afterward, but at first not so severely as usual; voices seem more natural; memory and sight better; appetite much better; less faintness; can better follow what people say; dreamy feeling and feeling of being somebody else less; the bad taste in mouth did not return after cleansing the teeth.

June 30th, 1.30 P. M. Rather weaker from over-exertion; less giddy; no feeling of opening and shutting in head; voices seem nearly natural; appetite much better, has eaten meat; feels faint; bad taste in mouth *only* on waking; time does not seem so prolonged; has a new symptom (effect of *Cann.?*), a dull, stupid pain in the head. The latest symptoms, as before stated, are (*cœteris paribus*) of the greatest diagnostic value, and in selecting a second remedy, they are of the utmost value. Is a new remedy, therefore, to be chosen according to this new symptom, or the same remedy allowed to act? Hahnemann teaches (Organon, 249–51) that if a medicine produces *new* symptoms not appertaining to the disease, it is a proof that it is not perfectly homœopathic, and that another remedy must be selected. In the "*Chronic Diseases*," he also states that new symptoms may also arise from a perfectly homœopathic remedy, if the dose be too large. In the latter case, of course, the remedy should be allowed to act, without change or repetition, unless the new symptoms are so violent as to require an antidote. But how are we to distinguish? In this way: if the patient is generally much better, it is a proof that the remedy was homœopathic, and that therefore the new symptoms (unless excited by some accidental cause) were produced by the magnitude of the dose, or by a peculiar susceptibility to the pathogenetic action of the drug; and the action of the remedy, under these circumstances, should be in no way interfered with. If, however, there is not much improvement, it is a sign that the medicine was not truly homœopathic, in which case a new remedy must be selected with especial reference to the new symptoms. On this occasion, as the patient was better (not to mention the fact that the new symptom was of too vague a character to be diagnostic), I neither repeated the dose nor changed the remedy, but allowed the one dose to act.

July 2d. Yesterday much better in every respect, felt nearly well (thus verifying the accuracy of Hahnemann's teaching); to-day weaker from over-exertion of yesterday; no giddiness for last two days; memory still bad; appetite good, enjoys meat; felt faint to-day; can follow conversation better; time does not seem as prolonged; not so many mistakes when writing; is stronger than when I first saw her. Has the following new symptom: On waking to-day, great pain as if weight were on right temple, making her lie down, lasting all day, off and on; at times cold feeling, beginning in nape of neck and going down back, followed at once by general heat (this latter symptom she has had when she has caught cold, but this has not been the case on this occasion); sight unchanged; no other symptoms. The patient being generally better, and the stirring up of old symptoms being a good sign (see Hahnemann's *Chronic Diseases*), I still allowed the one dose to act.

July 9th. From 3d to 6th felt almost well; since that has had the following symptoms: Weight on right temple, drawing head backward, and compelling her to lie down, but to a less extent than before; also, pain all over forehead, and shooting pain going from right temple to left; all these head symptoms aggravated by noise; the dull, stupid pain in head has returned for the last three days; feels much stronger; memory good; apparent duration of time nearly natural; sight a little better; sleep not quite so good for three days; the dreamy state, with inability to follow conversation, has returned for the last two days, but is less than before; no other symptoms. As she was still improving, and the recurring symptoms (weight in temple, dreamy state, etc.,) were less severe than at first, I still allowed the one dose to act in spite of the new symptoms (the drawing backward of head, and shooting from right to left temples); had these, however, been as severe as before, and proved persistent, it would have indicated that the remedy had now done all it could, and that a fresh medicine must be selected to meet the new symptoms.

July 13th. Feels much stronger; sight better; sleep good; has still the dreamy feeling at times, but less (as this was one of the earliest symptoms to appear, so it was the last to disappear, as Hahnemann teaches); all the other symptoms gone since 10th.

July 24th. Feels much stronger; all the above symptoms gone, except the dreamy feeling, which still exists, at times, to a slight extent.

*Acid. Muriaticum in Diarrhœa.*—June 22d, 1870. Mr. —— had diarrhœa this afternoon, the weather being hot; stools watery, dark brown, preceded by uneasy pains in abdomen; during stools, smarting at anus; has had three stools in ninety minutes, the last two in twenty minutes; is very anxious to get cured, as he is going out this evening.

*Diagnosis of the remedy*, according to Bell's Repertory.—Smarting during stool: *Agar.*, *Chin.*, *Kali c.*, *Mur. ac.* Watery stools: *Agar.*, *Mur. ac.*, and many others which have not the preceding symptoms.

The symptoms of the stool failed to differentiate further, neither *Agar.* nor *Mur. ac.* having "diarrhœa from hot weather;" I, therefore, referred to the collective of aggravations of *any symptom in the body*, as given in Bœnninghausen's "*Pocket Book.*" Here I found that *Mur. ac.* has "aggravation from warmth," but not *Agar.* I

gave the patient, at once, a dose of *Acid. mur.* 3000 (Jenichen), and some more globules to be taken if the diarrhœa returned. He had no diarrhœa after this one dose. Next morning he took all the remaining globules at one dose, and had no further stool till 27th. He says this cure convinced him of the truth of Homœopathy more than anything he had yet seen. This case is another proof of the absolute necessity of a *collective* of conditions in a repertory, such as was adopted by Bœnninghausen, and followed in my own *Eye Repertory.*

*Nat. mur., Canth., Rhus and Merc. in Gleet.*—1870, October 6th. Mr. —— had gonorrhœa two years ago; it was treated allopathically, and a gleet remained. He then used injections, which stopped the gleet for three or four days only. Afterwards he used stronger injections of *Argentum nitricum,* which caused great pains, chordee and the formation of three lumps in urethra, which subsequently became one. He used a catheter every day for six weeks, after which the lump disappeared. Since then he has had gleet at times, sometimes lasting three months at a time. Inguinal glands hard and enlarged ever since the gonorrhœa. Has only once had gonorrhœa, and never syphilis.

*Present Symptoms.*—Slight milky discharge since July; uncontrollable urging to urinate every two or three hours for three days (this first appeared after the *Arg. nit.* injection, and then came on every half hour for six weeks); urinates only a little at a time; slight uneasiness at end of urethra on walking; itching in urethra during urination; if he attempts to hold the urine all the muscles of the body feel tense, and again relax when urine is passed; when the urging comes on, he cannot hold it more than three or four seconds; has to rise every night to urinate; drinking alcohol increases the gleet; inguinal glands hard and enlarged; injections of arrow-root stop the discharges but increase the itching.

*Diagnosis of the remedy.*—Itching when urinating: *Ambr., Graph., Lycop., Mez., Nat. mur., Nux, Sars., Thuya.*

Urging to urinate little and often: *Nat. mur., Sars.,* and many others which have not the itching.

As *Nat. mur.* is an antidote to *Arg. nit.,* I gave one dose of 1000 (Jenichen).

October 13th. Improved next day. Uneasiness, itching and tension all gone; less discharge and urgency; has only once had to rise to urinate; can hold urine easily for four hours; glands unchanged; can now drink sherry and porter without increasing the gleet.

October 22d. Discharge the same; urging nearly gone, but increased by wine or beer; has not had to rise to urinate. Since the dose, stool more scanty than usual (effect of *Nat. mur.?*); glands swollen, no pain in them, even on violent exercise; alcohol increases the gleet, but to a less extent than before.

October 31st. Urging less, not increased by wine; stool as before; glands in right groin are natural, on left swollen; can easily hold urine six hours; discharge for last three days rather increased and more *sticky; when urinating smarting in urethra*, about an inch from end of penis; for a few days stream of urine double. As the improvement seemed to have nearly ceased, and new important symptoms arisen, a new remedy had to be selected.

*Diagnosis of the remedy.*—Stream double: *Arg. nit., Canth., Petr., Rhus.*

Pain during urination: *Arg. nit., Canth., Rhus* (and others).

Tenacious discharge: *Canth.*

One dose of *Cantharis* 1000 (Jenichen) was given.

November 11th. No urging; left inguinal gland rather painful on moving; discharge has been much better, but is now increased from indulgence in ale, wine and tobacco; smarting less severe and less often; stream not so often double.

November 19th. Smarting less; stream double at times; no pain in groin; discharge much more watery; feels a *hard swelling in urethra.*

*Diagnosis of the remedy.*—Swelling of urethra is found under *Canth., Merc., Nit. ac., Rhus.*

Stream double: *Canth., Rhus.*

As *Canth.* had been given before, and had now apparently ceased its action, one dose of *Rhus.* 2000 (Jenichen) was given.

November 26th. On 20th and 21st discharge increased (had drunk spiced ale), since then much less, has stopped entirely at times; no smarting since 21st; stream not double; pain at times in groin on walking; during the week has taken more wine than usual and smoked, but is nevertheless better.

December 3d. Discharge unchanged; for a few days *smarting on beginning to urinate*, in the urethra, near the glands; stream double for the last week; does not feel the lump; three days ago *penis felt very hot to the touch*, but not subjectively.

*Diagnosis of the remedy.*—Pain on beginning to urinate: *Canth., Caust., Clem., Merc.*

Heat of penis: *Caust., Canth., Merc.*

As *Canth.* had been given before, the choice was reduced to *Caust.* and *Merc.* Here the *anamnesis* proved of value; *Merc.* alone has the swelling in urethra, and this symptom, though it no longer existed, formed an element in the case. One dose of *Merc. viv.* 200 (Lehrmann) was given.

December 10th. Discharge has ceased at times; is no worse to-day in spite of drinking all kinds of wines last night, and dancing from 9 P. M. to 4 A. M.; the smarting went but returned to-day, lasting nearly all the time of urination (from the wine?); stream not double; stiffness in groins, at times, when walking fast.

December 17th. Discharge less, ceases at times; smarting less; stream doublé at times, if bladder is not full; groins as before, still some swelling of the glands.

December 29th. Discharge very slight; no smarting; stream not so double; groins better. The patient considered himself cured, and did not return.

This case proves the following points: 1st. The evil effects of injections, even those which, by reason of their power to inflame the urethra, have been by certain physicians considered homœopathic to the disease. 2d. That though the patient indulged in alcohol, tobacco and sexual intercourse as much as ever, single doses of high potencies were sufficient.

